# Downregulation of miR-23b by transcription factor c-Myc alleviates ischemic brain injury by upregulating Nrf2

**DOI:** 10.7150/ijbs.61399

**Published:** 2021-08-26

**Authors:** Rui Xin, Danhua Qu, Shuang Su, Bin Zhao, Dawei Chen

**Affiliations:** 1 Jilin University, Changchun 130000, P. R. China.; 2 Department of Radiology, the Second Hospital of Jilin University, Changchun 130000, P. R. China.; 3 Department of Respiratory and Critical Diseases, the Second Hospital of Jilin University, Changchun 130000, P. R. China.; 4 Sinopec Research Institute of Safety Engineering, Qingdao 266000, P. R. China.; 5 Department of Neurosurgery, the Second Hospital of Jilin University, Changchun 130000, P. R. China.; 6 Department of Radiation Protection, School of Public Health, Jilin University, Changchun 130000, P. R. China.

**Keywords:** Ischemic brain injury, c-Myc, microRNA-23b, Nuclear factor erythroid 2-related factor 2, Middle cerebral artery occlusion

## Abstract

Ischemic brain injury (IBI) is a common acute cerebral vessel disease that occurs secondary to blockage in arteries, mainly characterized by insufficient blood supply to the brain. The transcription factor c-Myc in IBI continues to be implicated in numerous studies. This study was conducted with emphasis placed on the underlying mechanism of c-Myc in IBI. Clinical samples were collected from IBI patients. Middle cerebral artery occlusion (MCAO) was induced in mice by inserting a suture from the external carotid artery to the anterior cerebral artery through the internal carotid artery to mechanically block the blood supply at the origin of the middle cerebral artery, and cortical neurons from mice were exposed to oxygen glucose deprivation (OGD) conditions for IBI model *in vitro* construction. RT-qPCR was performed to determine microRNA-23b (miR-23b) expression. TUNEL staining and Western blot analysis was conducted to detect apoptosis. The regulatory relationship was analyzed by dual-luciferase reporter gene assay. After loss- and gain-of-function assays, triphenyltetrazolium chloride staining was carried out to detect the area of cerebral infarction, after which the spatial memory in mice was evaluated with Morris water maze test. As per our findings, miR-23b was upregulated in the serum of IBI patients and OGD-treated murine primary neurons. Silencing of miR-23b resulted in reduced OGD-induced neuronal apoptosis. miR-23b inversely targeted nuclear factor erythroid 2-related factor 2 (Nrf2) and c-Myc negatively regulated miR-23b expression. Overexpression of c-Myc and inhibition of miR-23b led to reduced neurological scores, infarction area, neuronal apoptosis, shortened platform arrival time and significantly increased the time spent on the platform quadrant and the times of crossing the platform* in vivo*. Collectively, downregulated miR-23b by c-Myc might alleviate IBI by upregulating Nrf2.

## Introduction

Ischemic brain injury (IBI) is a common acute cerebrovascular disease that develops as a consequence of insufficient blood supply to the brain 1. Cerebral ischemic injury is a leading cause of premature death and physical disability with a high morbidity, which has been the source of wide-ranging effects on individuals, including mental financial burdens 2. Based on a previous study, the significant reduction in mortality caused by acute cerebral ischemia hasn't been reciprocated when it comes to the incidence of accompanied ischemic stroke 3. Cerebral ischemia contributes to neuronal cell death and cerebral infarction or even ischemic stroke, thus causing severe morbidity and high mortality, especially for diabetic patients 4. Patients with cerebral ischemia present with neurological deficit, such as cognitive and severe movement disorders 5. Cerebral ischemia is detrimental to the adaptive and innate immune system 6 and leads to oxidative damage 7, inflammation 8, and neuronal apoptosis 9, 10. Therefore, research at this stage is a high priority as to how to alleviate ischemic injury to the brain. However, the main challenge in the development of treatment options for IBI is the delivery of therapeutic molecules to ischemic sites 11.

Transcription factors are regulatory proteins that serve as regulators of gene expression and cell fate 12. The transcriptional factor c-Myc is a major oncogene with a variety of transcriptional functions, including regulating cell cycle, proliferation and apoptosis 13. c-Myc has been found to be involved in the glycolytic-mitochondrial regulation in ischemic injury 14. In addition, prior studies have thoroughly demonstrated the relationship between c-Myc and microRNAs (miRNAs). For example, c-Myc could inhibit miR-23a/b and resulted in elevation of mitochondrial and glutamine metabolism which is regulated by miR-23a and miR-23b 15. There exists a strong correlation between miRNAs and ischemia induced brain injury 16. For instance, miR-15a is associated with IBI 17 and miR-124 is involved in protecting against focal cerebral ischemia 18. Patients with ischemic stroke and transient ischemic attack presented with high serum levels of miR-23b-3p 19. However, the mechanism by which miR-23b regulates IBI remains unclear. Microarray-based analysis identified nuclear factor erythroid 2-related factor 2 (Nrf2) as a potential downstream target gene of miR-23b. Nrf2 has neuroprotective effects on cerebral ischemic injury 20. In addition, there exists extensive evidence confirming that Nrf2 exerts a significant effect on the recovery of permanent IBI 21. As the major regulatory factor of genes that protects cells, it also prevents carcinogenesis 22. The activation of Nrf-2 pathway alleviates the oxidative stress and inflammation following IBI 23. Cashew nuts can alleviate ischemia/reperfusion injury by inducing Nrf2 expression 24. In the present study, we aim to explore the mechanism underlying c-Myc involvement in IBI, which provides a significant tool in the study revolving around IBI and aid the development of a potential cell therapy for IBI.

## Materials and Methods

### Ethics statement

The protocol of clinical sample collection was approved by the Ethics Committee of the Second Hospital of Jilin University and was conducted in strict accordance with the *Declaration of Helsinki*. All the patients provided their written informed consent. All animal experiments were conducted following the standard of *the Guide for the Care and Use of Laboratory animals* published by the National Institutes of Health.

### Bioinformatics prediction

TransmiR v2.0 (http://www.cuilab.cn/transmir) was used to identify the miR-23b-related transcription factors, from which neuro-related factors were selected. Following the analysis of the scores of potential candidates, the possible transcription factors were predicted. The downstream target genes of miR-23b were predicted by the microT (http://diana.imis.athena-innovation.gr/DianaTools/index.php?r=microT_CDS/index), StarBase (http://starbase.sysu.edu.cn/), RNAInter (http://www.rna-society.org/rnainter/) and miRDB databases (http://www.mirdb.org/). The intersection of the results obtained from the 4 databases was determined to increase the confidence level of prediction. The interaction analysis was performed on the STRING (https://string-db.org/) with 0.4 as the lowest interaction score. The IBI-related GSE22255 dataset was downloaded from the Gene Expression Omnibus database (https://www.ncbi.nlm.nih.gov/geo/) loaded with annotation file GPL570, including 20 normal samples and 20 IBI samples. Differential analysis was performed to screen out significantly highly expressed genes using the R language “limma” package (http://www.bioconductor.org/packages/release/bioc/html/limma.html) with logFoldChange > 1.2 and *p* value < 0.005 serving as the threshold.

### Collection of clinical samples

Forty patients (26 males and 14 females; aged 41-82 years with a mean age of 59.65 ± 9.59 years) hospitalized due to IBI between May 2016 and October 2018 at the Second Hospital of Jilin University were included in our study. All patients were attacked by IBI within 24 h for the first time. In the classification standard of Trial of Org 10172 in Acute Stroke Treatment, all patients with non-cardiogenic IBI induced by atherothrombotic, lacunar or other unknown causes were excluded in accordance with findings obtained by computed tomography and magnetic resonance imaging. Patients with severe hepatic and renal insufficiency, autoimmune disease, craniocerebral injury and cerebral hemorrhage, brain tumor, blood disease, preceding infection or thyroid disease were also excluded. Meanwhile, 25 healthy individuals (15 males and 10 females; aged 49 - 76 years with a mean age of 65.56 ± 6.29 years) were selected as the normal controls. A total of 3 mL venous blood was collected from all fasting participants. Blood from patients with IBI was obtained the next morning after hospitalization and those from healthy participants were taken on the day the participants underwent medical examination. After the venous blood was centrifuged at 1500 ×g for 20 min at 4 °C, the supernatant was extracted, placed in a centrifuge tube and kept in a refrigerator at -80 °C for subsequent experiments.

### Culture and transfection of primary neurons extracted from mice

According to a previously described method 25, the embryo was removed from the postnatal day 17-18 (P17-P18) mice (irrespective of gender), after which the brain tissues were taken out completely. The blood vessels and meninges were discarded carefully in the pre-cooled phosphate-buffered saline (PBS) and the brain tissue was rinsed 2-3 times. The brain tissues were cut repeatedly with ophthalmic scissors, transferred to a culture dish and detached with TrypLE Express Enzyme (#12604013, Thermo Fisher Scientific, Waltham, MA, USA) and DNaseI (0.5 mg/L) (#AMPD1, Sigma-Aldrich Chemical Company, St Louis, MO, USA) at 37 °C for 20 min. Next, the detached single cell suspension was filtered with a 70 μM cell sieve, then seeded into neurobasal medium with B27 supplement (#117504044, Thermo Fisher Scientific) and cultured at 37 °C under 5% CO_2_ and 95% air. The culture dish was pre-embedded with poly-D-Lysine (#A-003-E, Millipore, Billerica, MA, USA) and Laminin (#23017015, Thermo Fisher Scientific) and the medium was renewed half every other day.

Primary cortical neurons were exposed to oxygen glucose deprivation (OGD) conditions after *in vitro* culture for 12-14 days and infected using lentiviral vector LV5-green fluorescent protein (GFP) for gene overexpression (#25999; LV-GFP was a gift from Elaine Fuchs Addgene, Cambridge, MA, USA) and pSIH1-H1 copGFP for silencing of gene (LV601B-1; System Biosciences, Palo Alto, CA, USA). All used lentiviral vectors were provided by Genechem (Shanghai, China). Meanwhile, cells received treatment with lentiviral vector expressing negative control (NC) mimic (LV-NC), NC inhibitor (LV-Anti-NC), miR-23b (LV-miR-23b), miR-23b inhibitor (LV-Anti-miR-23b), or overexpressed c-Myc (LV-oe-c-Myc) or remained untreated (blank). The OGD-treated cells were infected with LC-Anti-NC, LC-Anti-miR, LV-oe-NC + LV-NC, LV-oe-Nrf2 + LV-NC, LV-oe-Nrf2 + LV-miR-23b or remained untreated (OGD). When the 293T cells reached 90% cell confluency, the viral packaging plasmids (REV, VSVG and PMDL) were co-transduced with the corresponding knockdown plasmids using calcium phosphate transfection for 8 h. The old medium was renewed with a fresh medium containing 1 mM sodium butyrate and the culture continued for 48 h. The virus liquid was collected for subsequent experiments 26. Lentivirus was packaged in 293T cells which were cultured in a complete medium of Roswell Park Memorial Institute-1640 containing 10% fetal bovine serum and were subcultured every other day. Cortical neurons were then added with lentivirus (1 × 10^8^ TU/mL) for infection, followed by OGD treatment according to instructions established in a previous study 27. Briefly, the neurons were gently washed three times with PBS, and the sugar-free Dulbecco's modified Eagle's medium preheated to 37 °C was added into culture medium which was cultured in an anaerobic incubator (95% N_2_ and 5% CO_2_) for 2 h at 37 °C. Then, the neurons were collected for subsequent experiments. The sequence of miR-23b was 5'-CUCAGGUGCUCUGGCUGCUUGGGUUCCUGGCAUGCUGAUUUGUGACUUAAGAUUAAAAUCACAUUGCCAGGGAUUACCACGCAACCACGACCUUGGC-3' and that of anti-miR-23b was 5'-GAACCCAGCACCAGACCCTGA-3'.

### Establishment of IBI mouse models by middle cerebral artery occlusion (MCAO)

A total of 105 C57BL/6 mice weighing 22-25 g and aged 8-10 weeks were purchased from Junke Biological Co., Ltd (Nanjing, Jiangsu, China) to establish the MCAO-induced models. The mice were kept in the day/night cycle for about 4 days with the free access to food and water. Then, mice were anesthetized with intraperitoneal injection of 60 mg/kg sodium pentobarbital (Rhone Merieux Company, Pinkenba, Queensland, Australia). Rectal temperature was maintained between 36.5 °C and 37.5 °C throughout the operation using a rectal thermostats probe and a heating pad with thermostatic regulation (FHC, Bowdoinham, ME, USA). After anesthesia, right common carotid artery and external and internal carotid arteries of mice were exposed to the air and the common carotid artery was ligated with sutures. A 7/0 silicone monofilament nylon suture with a diameter of 0.22-0.23 mm was introduced gently into the internal carotid artery through the external carotid artery and was pushed in up to 10 ± 0.5 mm until the round head reached the entrance of the right middle cerebral artery with slight resistance. MCAO modeling was verified with the use of a Laser Doppler flowmetry (PeriFlux 5000, Jarfalla, Sweden) (Figure S1). Regional cerebral blood flow was monitored during IBI was monitored with the use of laser Doppler flowmeter (MBFSD, Moor Instruments Ltd., Devon, UK). A 2 mm diameter borehole was made at a position of 1 mm above the skull and 5 mm on the side of bregma using a stereoscopic positioning device (SR-6n, Narishige Scientific Instrument Laboratory, Tokyo, Japan) and a low-speed dental drill. A needle laser probe was placed on the dura mater away from the visible cerebral blood vessels. Baseline homeostatic values were recorded before MCAO. Right femoral artery was intubated with a polyethylene tube to monitor arterial blood pressure and heart rate (Powerlab/16 Data Acquisition System, AD Instruments Pty Ltd., Mountain View, CA, USA).

The participant mice were divided into several groups consisting of 15 mice in each group, including the sham-operation, MCAO, right intracerebroventricular injection of LV-Anti-NC, LV-Anti-miR-23b, LV-oe-NC + LV-NC, LV-oe-c-Myc + LV-NC or LV-oe-c-Myc and LV-miR-23b in the ventricle 1 h after MCAO. Every 10 live mice were randomly selected from each group for the subsequent experiments.

Stereotactic injection was conducted as follows: mice were injected with lentiviruses 1 h after MCAO modeling 27, 28 and anesthetized with 3% pentobarbital sodium *via* intraperitoneal injection. Intraventricular injection of lentivirus (4 µL, 1 µL/min with titer of 2 × 10^8^ infectious-focus units [IFU]/mL) was performed using brain stereotaxic instrument (KOPF, Tujunga, CA, USA) and stepper-motorized microsyringe (Hamilton Bonaduz, Swithzerland), keeping the needle inserted for 5-10 min 27.

### Chromatin immunoprecipitation (ChIP) assay

Following manufacturer's protocol of EZ-Magna ChIP kit (EMD Millipore, Burlington, Vermont, USA), mouse cerebral cortical neurons were fixed with formaldehyde for 10 min to produce DNA-protein cross-linking. The sonicator (UP-250, Scientz, Ningbo, China) was set to disrupt cells and sonicate DNA into fragment of 200-500 bp for 10 s at an interval of 10 s with 15 cycles. The dimension of DNA was verified through gel electrophoresis. The fragmented chromatin solution underwent incubation with protein G beads at 4 °C for 2 h, followed by centrifugation at 12000 rpm at 4 °C for 10 min. The supernatant was collected and placed into 2 tubes, which were respectively incubated with NC immunoglobulin G (IgG) (ab109489, 1:300, Abcam Inc., Cambridge, UK) and c-Myc antibodies (1 : 50, #13987, Cell Signaling Technology Inc., Beverly, MA, USA) at 4 °C overnight. DNA-protein complex was precipitated using Protein Agarose/Sepharose on the next day. After centrifugation for 5 min at 12000 ×g, cell supernatant was discarded and the non-specific complex was eluted using ChIP wash buffer and de-crosslinked at 65 °C overnight. The DNA fragments were extracted and purified by phenol/chloroform, while the binding of c-Myc to miR-23b was detected using RT-qPCR on the miR-23b promoter.

### Dual-luciferase reporter gene assay

The 3'untranslated region (3'UTR) dual luciferase reporter carrier of Nrf2 and mutant plasmids including PmirGLO-Nrf2-wild type (WT) and PmirGLO-Nrf2-mutant type (MUT) at the binding site of miR-23b were constructed respectively. The reporter plasmid miR-23b mimic and NC plasmids were co-transfected into 293T cells, respectively. After transfection for 24 h, the 293T cells were lysed, and centrifuged at 12000 rpm for 1 min, with the supernatant collected. Dual Luciferase Reporter Assay System (Dual-Luciferase^®^ Reporter Assay System, E1910, Promega Corporation, Madison, WI, USA) was conducted to detect luciferase activity. Then, 100 µL firefly luciferase working fluid and 100 µL renilla luciferase working fluid were added to each cell to detect firefly luciferase and renilla luciferase which were used as relative luciferase activities.

### RT-qPCR

Total RNA was extracted from tissues or cells of 4 mice in each group using TRIZOL reagent (Invitrogen, Carlsbad, CA, USA). The primers for miR-23b and Nrf2 were provided by Invitrogen (Table S1). The total RNA was reversely transcribed into complementary DNA based on the instructions of different RT kits such as TaqMan™ MicroRNA Reverse Transcription Kit (4366596; Thermo Fisher Scientific) and High-Capacity cDNA (4368813; Thermo Fisher Scientific). RT-qPCR experiments were performed on ABI7500 qPCR instrument (Thermo Fisher Scientific) the SYBR^®^ Premix Ex Taq^TM^ (Tli RNaseH Plus) kit (RR820A,TaKaRa, Tokyo, Japan) on a real-time fluorescent quantitative PCR instrument (ABI, Foster City, CA, USA) with the 25 µL reaction system. The PCR reaction conditions were as follows: pre-denaturation at 95 °C for 5 min, 40 cycles of denaturation at 95 °C for 10 s, annealing at 60 °C for 20 s and extension at 72 °C for 20 s, and extension at 78 °C for 20 s. Glyceraldehyde-3-phosphate dehydrogenase (GAPDH) (Invitrogen) was utilized as internal reference for Nrf2 and U6 (Invitrogen) for miR-23b. The relative expression was analyzed using 2^-ΔΔCt^ method. ΔΔCt = (mean Ct value of the target gene of the experimental group - mean Ct value of GAPDH of the experimental group) - (mean Ct value of the target gene of the control group - mean Ct value of GAPDH of the control group).

### Western blot analysis

Total protein of cells or tissues was extracted using the efficient radioimmunoprecipitation assay lysis buffer (R0010, Solarbio Science & Technology Co., Ltd., Beijing, China) in strict accordance with the instructions. Then, the cells were lysed at 4 °C for 15 min and centrifugation was carried out for 15 min at 15000 r/min. The protein concentration of each sample was measured by a bicinchoninic acid kit (20201ES76, Shanghai Yeasen BioTechnologies Co., Ltd., Shanghai, China). The protein was separated by polyacrylamide gel electrophoresis and transferred to the polyvinylidene fluoride membrane. The membrane was blocked with 5% bovine serum albumin at room temperature for 1 h, and then incubated with diluted primary rabbit antibodies (Abcam) against Nrf2 (ab137550, 1:1000), c-Myc (ab32072, 1:1000), cleaved caspase-3 (ab49822, 1:500), B-cell lymphoma-2 (Bcl-2) (ab182858,1:2000) and Bcl-2 associated protein X (Bax) (ab199677, 1:1000) overnight at 4 °C. Then the horseradish peroxidase-labeled goat anti-rabbit IgG (ab205718, 1:10,000, Abcam) was added into the membrane and incubated at room temperature for 1 h. The image was developed by the developer VILBER FUSION FX5 (VILBER LOURMAT, France). ImageJ 1.48u software (National Institutes of Health, Bethesda, USA) was used for protein quantitative analysis, and the ratio of gray value of each protein to that of GAPDH was indicative of relative protein expression.

### Flow cytometry

Annexin V-fluorescein isothiocyanate (FITC)/propidium iodide (PI) apoptosis assay kit (C1062M, Beyotime, Shanghai, China) was employed to detect neuron apoptosis. A total of 1 × 10^6^ cells/mL were washed twice with cold PBS and then resuspended in 195 µL Annexin V-FITC binding solution, followed by the addition of 5 µL Annexin V-FITC binding solution and 10 µL PI. The cells were incubated at room temperature under dark conditions for 15 min. Finally, a flow cytometer (FACSVerse/Calibur/AriaIISORP, BD, USA) was used to quantify cell apoptosis.

### Neurological deficit score

The neurological function in each group of mice was evaluated based on previous literature 29. After successful MCAO model establishment, 6 mice were randomly selected from each group for neurological function tests, including exercise tests (such as forelimb flexion, hind limb flexion, and head movement, scoring on a scale of 0-6), balance test (scoring on a scale of 0-6), as well as reflection and abnormal motion test (scoring on a scale of 0-2). Total scores 1-4, 5-9, and 10-14 indicated mild, moderate, and severe lesions, respectively. Neurological function was independently assessed by 3 investigators.

### 2,3,5-triphenyl tetrazolium chloride (TTC) staining

Mice in each group were euthanized at 24 h post MCAO modeling. The brain tissues were isolated and cut into 5 coronal sections (2 mm). Each section was stained in 2% TTC solution at 37 °C for 30 min. The infarcted area was pale while the non-infarcted area was brick-red. The infarcted area and the non-infarcted hemisphere were analyzed using the Image J software (National Institutes of Health).

### Hematoxylin-eosin (HE) staining

The brain tissues were cut into sections (4 μm) and stained with hematoxylin (5 min) and 5% eosin (3 min). Histopathological examination was performed under a microscope.

### Terminal deoxynucleotidyl transferase-mediated 2'-deoxyuridine 5'-triphosphate nick end-labeling (TUNEL) staining

The mouse brain tissues were isolated, fixed in 4% paraformaldehyde overnight and then paraffin-embedded. The embedded tissues were sectioned into 5 mm slices. A total of 5 pieces of slices were dewaxed, added with 50 µL of 1% proteinase K dilution in each slice, placed in an incubator at 37 °C for 30 min and added with 0.3% H_2_O_2_ methanol solution to eliminate endogenous peroxidase (POD) activity. Then tissue slices underwent incubation at 37 °C for 30 min at room temperature, followed by the addition of TUNEL reaction solution and incubation in a humid box at 37 °C for 1 h under dark conditions. A total of 50 μL of Converter-POD, and 2% diaminobenzidine developing solution was added in the slices, and were incubated at room temperature for 15 min. Under the microscope, cells were shown with brownish-yellow nuclei, followed by the supplement of distilled water to terminate the reaction. The cells were counterstained by hematoxylin and the reaction was terminated by distilled water. Next, the cells were dehydrated by gradient ethanol, transparentized by xylene and mounted with neutral gum. Under an optical microscope with 40-fold lens, 10 visual fields were randomly selected from each slice, and the cells with brownish-yellow nuclei were regarded as apoptotic cells, while the cells with blue nuclei were considered as healthy cells. The ratio of the number of brown cells/blue cells was determined by obtaining the mean value from different fields and was regarded as the apoptosis rate of the neurons.

### Morris water maze test

A circular tub (120 cm in diameter, 60 cm in height) was filled with opaque water and a circular platform (6 cm in diameter), which was immersed 1 cm below the surface of the water. Before the test, the mice were allowed to adapt to testing environment for 20 min. Hidden platform training was conducted for 6 consecutive days, with each training consisting of four trials. For each test, the mice were allowed to search the hidden platform during the 60 s test period. If mice did not reach the platform within the set time, the mice were guided to the platform by investigators. The probe test was performed 24 h after the completion of the training. During the test, the platform was removed and the performance of the mice was recorded for 60 s. Mouse performance was measured based on the latency time to reach the platform, the time spent in each quadrant, and the times crossed the platform area.

### Statistical analysis

Statistical analysis was conducted by SPSS 21.0 (IBM Corp., Armonk, NY, USA). Measurement data were expressed as mean ± standard deviation. Unpaired *t* test was performed for comparison between two groups of data with normal distribution and homogeneous variance. One-way analysis of variance (ANOVA) was conducted for multi-group data comparison, followed by Tukey's post hoc test. Data at different time points were compared by Bonferroni-correct repeated measures ANOVA. Statistical significance was signified by *p* < 0.05.

## Results

### miR-23b expression is upregulated in IBI

**Figure 1:**
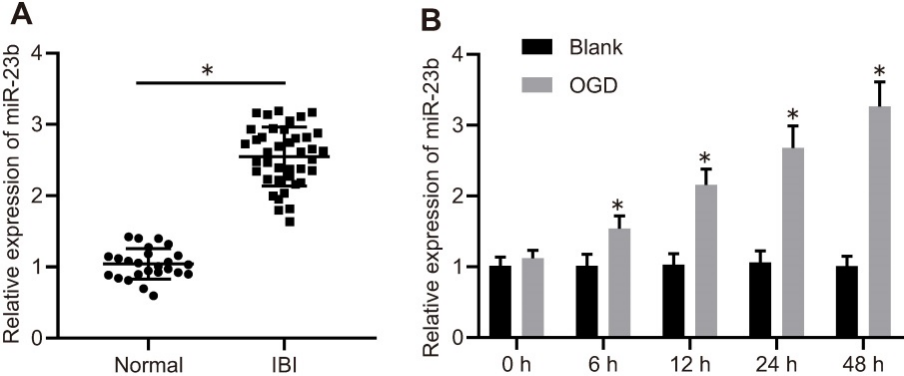
** miR-23b is highly expressed in IBI. A,** miR-23b expression in the serum of normal controls (n = 25) and patients with IBI (n = 40) determined by RT-qPCR. *p < 0.05 vs. the normal group. **B,** The expression of miR-23b in OGD-treated primary cortical neurons determined by RT-qPCR. *p < 0.05 vs. the blank group. The measurement data were expressed as mean ± standard deviation. Data between two groups were analyzed by unpaired t test. The experiment was repeated 3 times independently.

To explore the mechanism of miR-23b in IBI, miR-23b expression was detected in clinical samples with the application of RT-qPCR. The results showed that miR-23b was significantly increased in the serum of IBI patients compared with the normal control (Figure 1A). To further verify the expression of miR-23b in IBI *in vitro*, primary mouse cortical neurons were isolated from mice and cultured for 12-14 days, followed by exposure to OGD conditions. As shown in Figure 1B, RT-qPCR depicted that miR-23b expression was gradually elevated in OGD-treated neurons in a time-dependent manner. Collectively, high expression of miR-23b occurred in IBI.

### Downregulation of miR-23b alleviates neuron injury induced by OGD *in vitro*

**Figure 2:**
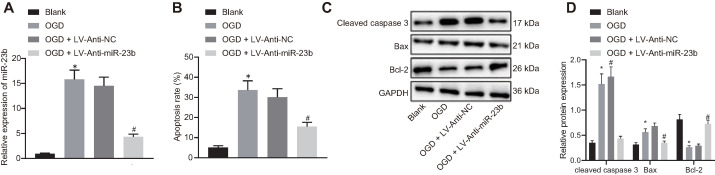
** Downregulated miR-23b alleviates OGD-induced neuron injury. A,** Inhibition effect of Anti-miR-23b on miR-23b expression in neurons detected by RT-qPCR. **B,** Apoptosis rate of neurons determined by flow cytometry. **C,** Protein bands of apoptosis-related proteins (cleaved caspase-3, Bax and Bcl-2) in neurons. **D,** Relative expression of apoptosis-related proteins (cleaved caspase-3, Bax and Bcl-2) in neurons measured by Western blot analysis. *p < 0.05 vs. the blank group; #*p* < 0.05 vs. OGD + LV-Anti-NC group. Measurement data were expressed by mean ± standard deviation. One-way ANOVA was conducted for multi-group data comparison, followed by Tukey's post hoc test. The experiment was repeated three times independently.

We then attempted to investigate the relationship between miR-23b and IBI* in vitro*. The results of RT-qPCR showed that the expression of miR-23b was significantly decreased in OGD-induced cells treated with Anti-miR-23b (Figure 2A). As an important form of neuron injury under OGD condition, apoptosis is associated with IBI. For exploration purpose, flow cytometry, displayed that apoptosis rate of neurons exposed to OGD conditions was increased significantly while miR-23b inhibition significantly decreased the apoptosis rate (Figure 2B). Western blot analysis data further revealed the upregulated apoptosis-related proteins Bax and cleaved caspase-3 as well as downregulated Bcl-2 in OGD-induced neurons, while miR-23b inhibition treatment brought about opposite results in OGD-induced neurons (Figure 2C and 2D). These results indicated that downregulation of miR-23b can relieve IBI *in vitro*.

### Downregulation of miR-23b inhibits nerve injury in MCAO mice

**Figure 3:**
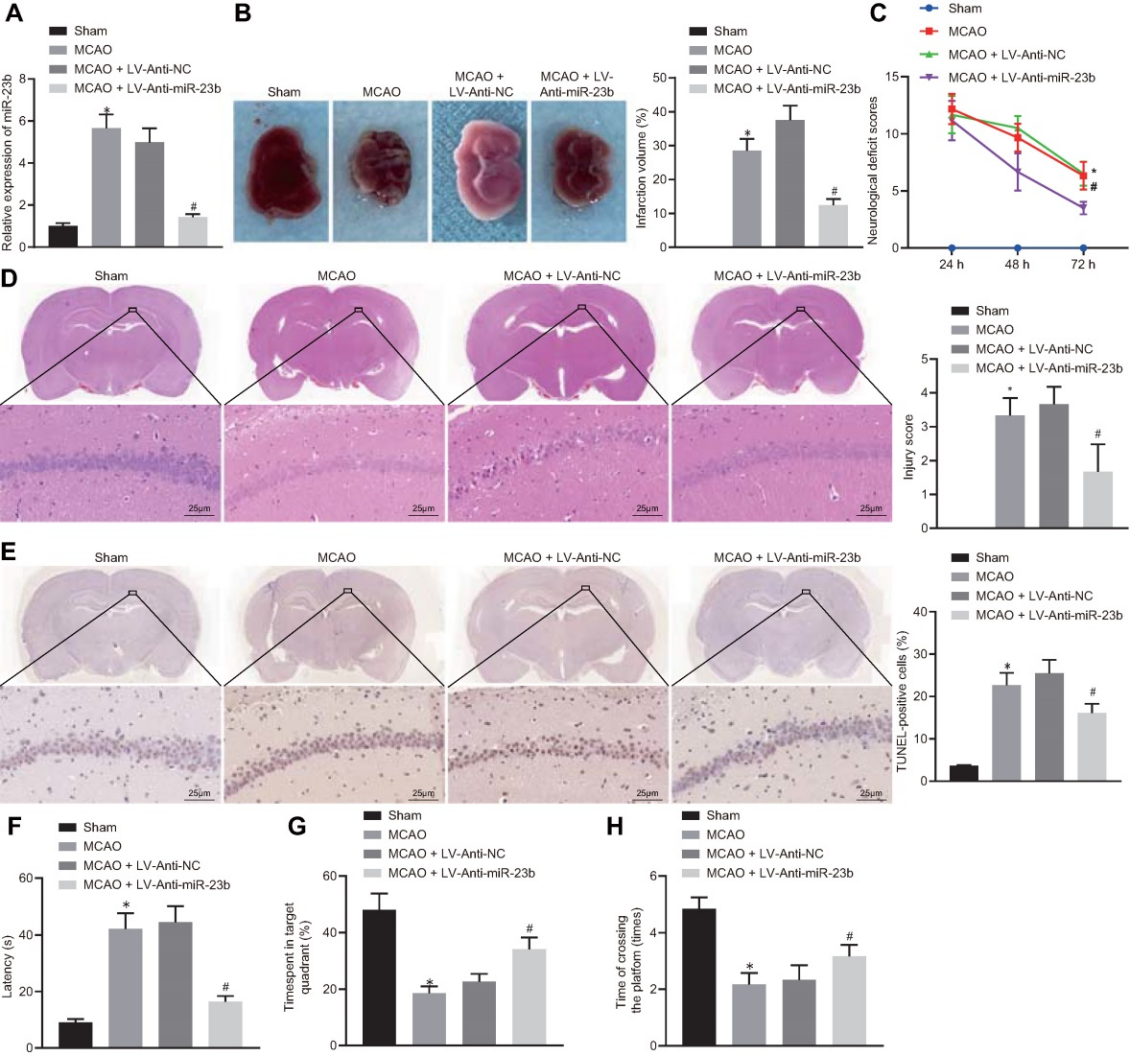
** miR-23b knockdown inhibits nerve injury in MCAO mice. A,** miR-23b expression in mouse brain tissues measured by RT-qPCR. **B,** Area of brain infarction in mice detected by TTC staining. **C,** Neurological scores of mice in each group. **D,** HE (400 ×) staining of brain tissues of mice. **E,** TUNEL (400 ×) staining of apoptosis in brain tissues of mice. **F,** Latency of mice examined by Morris water maze test. **G,** Time of mice spent in target quadrant examined by Morris water maze test. **H,** Times of mice crossing the platform examined by Morris water maze test. *p < 0.05 vs. the sham group; #*p* < 0.05 vs. MCAO + LV-Anti-NC group. Measurement data were expressed by mean ± standard deviation. One-way ANOVA was conducted for multi-group data comparison, followed by Tukey's post hoc test. Pairwise comparison of neurological scores in panel C was analyzed by non-parametric test Mann-Whitney test. n = 10.

To investigate the role of miR-23b in IBI in mice, a mouse MCAO model was first generated. RT-qPCR showed that miR-23b expression was significantly increased in brain tissues of MCAO mice compared with those from sham-operated mice. In contrast, miR-23b expression in brain tissues of MCAO mice was decreased following treatment with LV-Anti-miR-23b (Figure 3A). TTC staining results manifested that MCAO mice exhibited obvious cerebral infarction, and the inhibition of miR-23b reduced the area of cerebral infarction (Figure 3B). Neurological scoring results showed that MCAO mice presented with significantly increased neurological scores, while inhibition of miR-23b significantly decreased the neurological score of mice at 24, 48 and 72 h after MCAO model establishment (Figure 3C). HE and TUNEL staining assays displayed that a large number of neurons in hippocampal CA1 areas of mice were damaged after MCAO treatment as evidenced by shrank nuclear pyramidal neurons, chromatin condensation, increased cell death and apoptosis. However, inhibition of miR-23b significantly reversed these results (Figure 3D and 3E). On the 26th day after MCAO, the mice were trained for 4 consecutive days. On the 30th day, Morris water maze was implemented, which documented that in MCAO mice, the exploration time of the submerged platform was increased, the time spent in the safety quadrant was reduced, and the times that mice crossed the platform area were decreased. The results were reversed by the further treatment of the inhibition of miR-23b (Figure 3F-H). Collectively, downregulation of miR-23b suppresses nerve injury in MCAO mice.

### miR-23b promotes OGD-induced neuron injury by suppressing the expression of Nrf2

**Figure 4:**
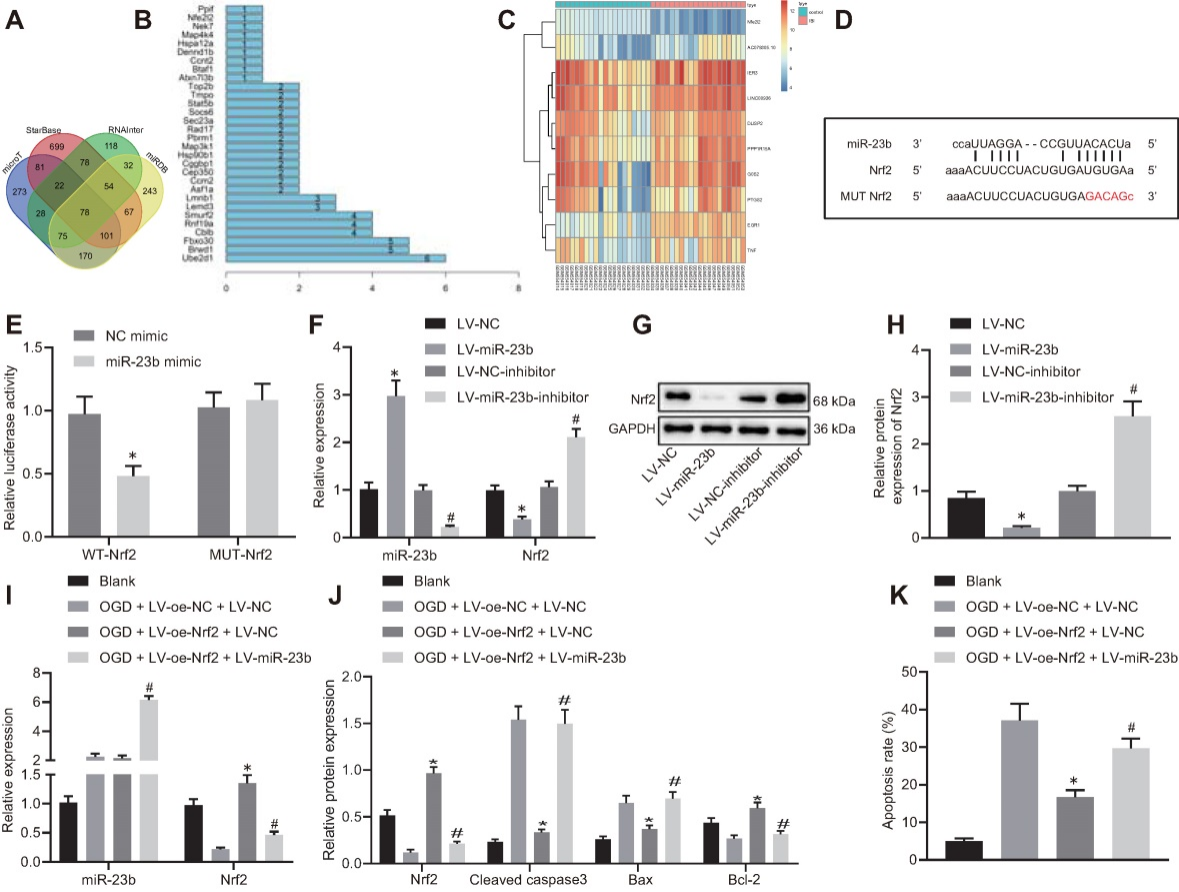
** miR-23b accelerates OGD-induced neuron injury through suppressing Nrf2. A,** Venn diagram displaying the prediction results on downstream target genes of miR-23b obtained from MicroT data (http://diana.imis.athena-innovation.gr/DianaTools/index.php?r=microT_CDS/index), StarBase database (http://starbase.sysu.edu.cn/), RNAInter database (http://www.rna-society.org/rnainter/) and miRDB database (http://www.mirdb.org/). **B,** Interaction analysis results of 78 target genes from STRING (https://string-db.org/). The X-axis indicated the number of genes that could interact with genes corresponding to the Y-axis, and the Y-axis indicated the gene name. **C,** Heatmap displaying top 10 differentially expressed genes from microarray database GSE22255. **D,** Binding sites between miR-23b and Nrf2-3'UTR. **E,** Dual luciferase reporter assay for detection of luciferase activity, *p < 0.05 vs. NC-mimic treatment. **F,** Expression of miR-23b and Nrf2 in mouse cerebral cortical neurons assessed by RT-qPCR. **G,** Western blots of Nrf2 in mouse cerebral cortical neurons. **H,** Nrf2 protein band in mouse cerebral cortical neurons detected by Western blot analysis. **I,** miR-23b and Nrf2 expression in mouse cerebral cortical neurons measured by RT-qPCR. **J,** Protein expression of Nrf2, Bax, Bcl-2, and cleaved caspase-3 in mouse cerebral cortical neurons detected by Western blot analysis. **K,** Flow cytometry for detection of neuron apoptosis in mouse cerebral cortex. In panels F-H: * *p* < 0.05 *vs.* LV-NC; # *p* < 0.05 vs. LV-NC-inhibitor. In panels I-K: * *p* < 0.05 *vs.* OGD + LV-oe-NC+LV-NC group; # *p* < 0.05 *vs.* OGD + LV-oe-Nrf2 + LV-NC group. Measurement data were expressed as mean ± standard deviation. Unpaired *t* test was performed for data comparison between two groups. One-way ANOVA was conducted for multi-group data comparison, followed by Tukey's post hoc test. The experiment was repeated three times independently.

With an attempt to investigate the downstream mechanism of miR-23b involved in IBI, we first predicted the downstream target genes of miR-23b using the microT, StarBase, RNAInter and miRDB databases. Following intersection analysis of the predicted results, 78 target genes were identified (Figure 4A). The STRING online website further showed that 30 genes of the 78 target genes had an interaction (Figure 4B). Differential analysis on the GSE22255 dataset revealed significantly lower expression of Nrf2 (Nfe2l2) in patients with IBI when compared with normal controls, while the expression of the rest 29 genes exhibited no significant difference in the GSE22255 dataset (Figure 4C). Therefore, we selected Nrf2 as the downstream target gene of miR-23b for subsequent use.

In addition to insignificant difference of 29 genes in the GSE22255 dataset, the binding site between miR-23b and Nrf2 was obtained through the StarBase database (Figure 4D). Meanwhile, dual-luciferase reporter gene assay exhibited that the luciferase activity of cells transfected with miR-23b mimic/WT-Nrf2-3'UTR was significantly decreased while no significant difference was observed in the luciferase activity of MUT-Nrf2-3'UTR (Figure 4E). In the cultured mouse cortical neurons, overexpression of miR-23b resulted in obviously diminished Nrf2 level, while inhibition of miR-23b contributed to notably increased Nrf2 level (Figure 4F-H). These results suggested that miR-23b targeted Nrf2 and suppressed its expression.

The role of miR-23b in IBI was further investigated by orchestrating Nrf2. RT-qPCR and Western blot analysis results exhibited that Nrf2 expression was decreased in OGD-induced mouse cortical neurons. However, following overexpression of Nrf2, Nrf2 expression was augmented strikingly, which was nullified by further miR-23b overexpression (Figure 4I). As reflected by Western blot analysis, overexpression of Nrf2 reduced Bax and cleaved caspase-3 levels, while elevated Bcl-2 level. Meanwhile, miR-23b overexpression reversed the trends (Figure 4J). Flow cytometric data displayed that overexpression of Nrf2 reduced the apoptosis rate of mouse cortical neurons, which was annulled by further overexpression of miR-23b (Figure 4K). These results demonstrated that miR-23b participated in IBI by inhibiting Nrf2 expression.

### c-Myc mitigates IBI in mice by manipulating miR-23b/Nrf2 axis

**Figure 5:**
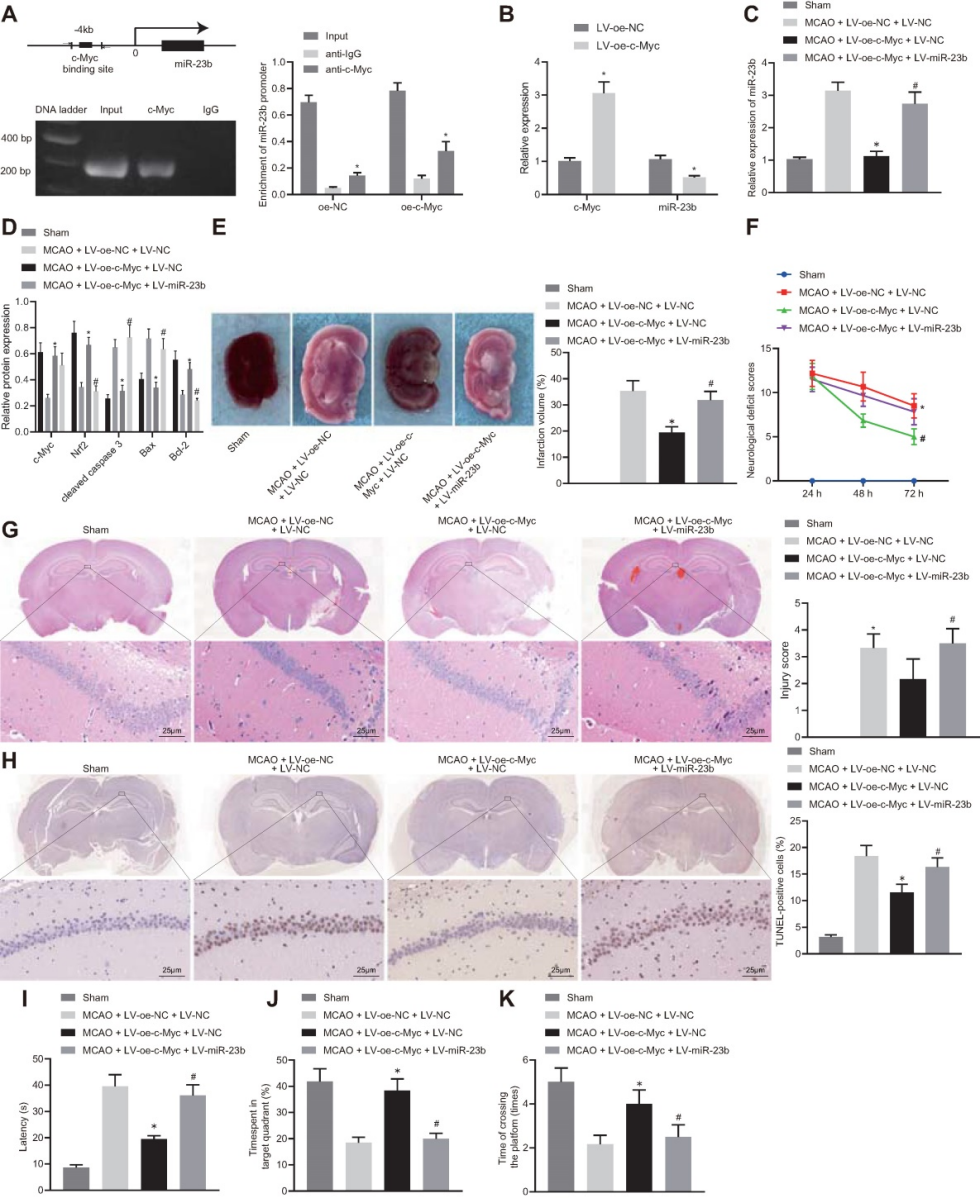
** c-Myc alleviates nerve injury in MCAO mice through regulating miR-23b/Nrf2 axis. A,** The binding site of c-Myc in miR-23b promoter and enrichment of transcription factor c-Myc in miR-23b promoter detected by ChIP. **B,** miR-23b expression in mouse cerebral cortex neurons detected by RT-qPCR. **C,** miR-23b expression in mouse brain tissues detected by RT-qPCR. **D,** Protein expression of c-Myc, Nrf2, Bax, Bcl-2, and cleaved caspase-3 in mouse brain tissues by Western blot analysis. **E,** Area of brain infarction was detected by TTC staining. **F,** Neurological scores of mice in each group. **G,** HE (400 ×) staining of brain tissues. **H,** TUNEL (400 ×) staining of apoptosis of brain tissues. I, Latency of mice examined by Morris water maze test. J, Time of mice spent in target quadrant examined by Morris water maze test. K, Times of mice crossing the platform examined by Morris water maze test. * *p* < 0.05 *vs.* the anti-IgG, LV-oe-NC, or MCAO + LV-oe-NC + LV-NC group, # *p* < 0.05 *vs.* the MCAO + LV-oe-c-Myc + LV-NC group. Measurement data were expressed by mean ± standard deviation. Unpaired *t* test was performed for data comparison between two groups. One-way ANOVA was conducted for multi-group data comparison, followed by Tukey's post hoc test. Pairwise comparison of neurological scores in panel F was analyzed by non-parametric test Mann-Whitney test. n = 10.

A series of experiments were conducted to understand the relationship among c-Myc, miR-23b and Nrf2 in IBI. The results of ChIP assay showed the specific enrichment of transcription factor c-Myc in the miR-23b promoter region of the mouse cerebral cortical cells (Figure 5A, Figure S2A, B). Overexpression of c-Myc resulted in the significant inhibition of miR-23b expression. In addition, according to previous reports 30, 31, we also excluded the possibility that c-Myc could directly bind to the Nrf2 promoter region to regulate its expression (Figure S3).

Based on the above studies, we proposed that c-Myc could attenuate IBI in mouse tissues by regulating the miR-23b/Nrf2 axis. RT-qPCR results manifested that overexpression of c-Myc resulted in a decrease in miR-23b expression in OGD-exposed cerebral cortical neurons (Figure 5B). RT-qPCR and Western blot analysis depicted that c-Myc and Nrf2 expression was potently lowered, miR-23b expression was markedly elevated in brain tissues of MCAO mice. However, overexpression of c-Myc led to enhanced Myc and Nrf2 expression and reduced miR-23b expression in brain tissues of MCAO mice, which was counteracted by further miR-23b overexpression (Figure 5C and D). Western blot analysis revealed that the brain tissues of MCAO-modeled mice presented increased Bax and cleaved caspase 3 levels and decreased Bcl-2 expression. Overexpression of c-Myc led to lowered expression of Bax and cleaved caspase-3 and augmented Bcl-2 expression in brain tissues of MCAO mice, which was abrogated by further miR-23b overexpression (Figure 5D).

Additionally, TTC staining results showed that highly expressed c-Myc in mice with MCAO resulted in a significant decrease in cerebral infarction area, while further overexpression of miR-23b increased cerebral infarction area in MCAO mice in the presence of overexpressing c-Myc (Figure 5E). Neurological scores displayed that overexpression of c-Myc reduced the neurological score at 24, 48 and 72 h after MCAO model establishment, while further overexpression of miR-23b reversed this trend (Figure 5F). HE and TUNEL staining assays showed that the neurons in hippocampal CA1 areas of MCAO mice sustained serious injuries as evidenced by atrophy of nuclear pyramidal neurons, chromatin condensation, and elevated cell death and apoptosis. However, overexpression of c-Myc attenuated neuronal injury and apoptosis significantly, while further miR-23b overexpression abrogated the attenuating effects of c-Myc elevation in MCAO mice (Figure 5G and 5H). On the 26^th^ day after MCAO, the mice were trained for 4 consecutive days. On the 30^th^ day, Morris water maze was conducted and the results showed that following c-Myc overexpression, mice spent less time exploring the submerged platform, and more time staying in the safety quadrant with increased times of crossing the platform area, which was nullified by further miR-23b overexpression (Figure 5I-K). Conclusively, downregulation of miR-23b can suppress IBI and that c-Myc alleviated IBI by downregulating miR-23b expression and increasing Nrf2 expression.

## Discussion

IBI is a disease with high prevalence associated with brain damage that has been the source of a widespread concern worldwide 32. The* in vivo* findings of IBI include severe functional defects and apoptosis of neurons 33. Despite the impressive breakthroughs regarding the potential mechanisms of IBI, therapeutic pathways for IBI are poorly understood 34. In the present investigation, we reported a novel signal axis of c-Myc/miR-23b/Nrf2 for alleviation of IBI.

In a previous review, upregulation of c-Myc expression was observed in rat brain tissues and could facilitating the recovery of motor function after ischemic stroke, which indicates that upregulation of c-Myc expression can alleviate IBI 35. This finding is consistent with our finding on the roles of c-Myc and its downstream mechanism in IBI. Interestingly, previous studies have indicated that c-Myc expression increases after MCAO 36 or hypoxia 37, 38 and elevated c-Myc exacerbates apoptosis via miR-23b suppression 38, which seems to considerably differ from the results obtained in the current study. However, Huang *et al.* have only determined the nuclear c-Myc level, which cannot represent c-Myc expression in the whole cell. As for study carried out by Greenway *et al.* and Chen *et al.*, samples were exposed to oxygen-deficient environment, which is similar to yet not exactly the same with ischemic condition. Hereby, we believe that our findings are not contrary to theirs.

Our results verified that miR-23b was upregulated in IBI and the downregulation of miR-23b expression relieved IBI while reducing neuronal apoptosis. Moreover, knockdown of miR-23b also contributed to attenuated IBI in mouse tissues* in vivo*. Consistent with our study, the inhibition of miR-23b resulted in the upregulation of murine double minute 4 in rats through p53 signaling pathway, thereby protecting against ischemia-reperfusion (IR) injury 39. The myocardial function disrupted by IR injury could be reversed following the suppression of miR-23b expression 40. The protein expression of Bax/Bcl-2 and cleaved caspase-3 was significantly suppressed as a result of miR-23b inhibition. Inhibition of miR-23b-3p significantly suppresses CHON-001 cells apoptosis caused by interleukin-1β by enhancing the levels of denatured type II collagen and aggregated protein cleavage and reducing the expression of Bax and active cystic caspase 3 41. These findings are consistent with our experimental results. Furthermore, rats that suffered from ischemic cerebral infarction induced by transient occlusion of blood to brain exhibit aberrant neuronal apoptosis 42, which is partially consistent with our findings suggesting that mice conducted with MCAO experiments showed neuronatrophy, increased neuronal apoptosis and our results further revealed that inhibition of miR-23b contributed to the evident decrease in neuronal injury and apoptosis. In addition, the sequences of miR-23a and miR-23b have been reported to be similar, with the exception of 1 nucleotide 43. Moreover, miR-23a has been demonstrated to mediate cardiomyocyte apoptosis by targeting manganese superoxide dismutase 44, which further highlights the need for further studies exploring the functional role of miR-23a in IBI.

Subsequent experiments found that miR-23b aggravated IBI through the inhibition of Nrf2 while c-Myc could reverse the IBI *via* the miR-23b/Nrf2. Nrf2 was predicted to be the target gene of miR-23b by microT data, StarBase database, RNAInter database and miRDB database. Nrf2 is an important signaling pathway for reducing myocardial infarct area and maintaining cardiac function that occurs secondary to myocardial IR injury 45. Inkgolides and bilobalide protect neurons from oxidative stress by upregulating antioxidant protein levels by activating Nrf2 signaling pathway 46. Ginsenoside Rg1 inhibits miR-144 activity, thereby upregulating Nrf2 expression, resulting in the reduction of oxidative stress after IR, and providing protection against IR-induced neuronal damage 47. All these findings suggest that Nrf2 is capable of effectively alleviating ischemia-induced injury. Knockdown of Nrf2 eliminated brahma-related gene 1 (Brag-1) mediated neuroprotection, suggesting that Brag-1 attenuates OGD/reoxygenation-induced neuronal apoptosis through the upregulation of Nrf2 expression 48. In addition, Probucol inhibits inflammation and neuronal apoptosis that occurs secondary to spinal cord injury by activating Nrf2 signaling pathway 49. The aforementioned findings revealed that upregulated Nrf2 exerts an effective role in protecting neurons, which is in line with our results demonstrating that Nrf2 plays a functional role in alleviating brain injury and overexpression of Nrf2 significantly reduces the apoptosis rate of neurons. Consequently, this study revealed that the transcription factor c-Myc alleviated IBI by impairing the miR-23b-targeted inhibition of Nrf2. Notably, different reports have highlighted the direct effect of c-Myc on Nrf2 expression 30, 31; however the current study was unable to investigate this due to time and funding limitations and futures studies centered around this concept are encouraged.

**Figure 6:**
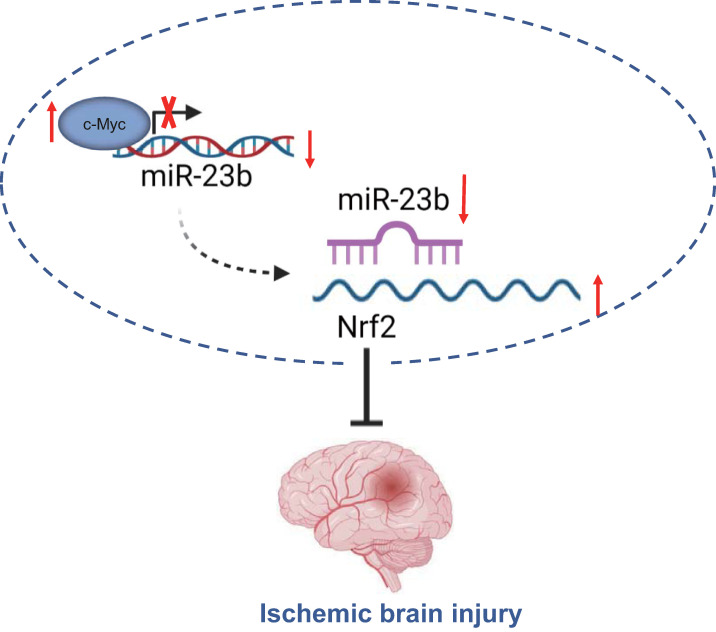
** c-Myc/miR-23b/Nrf2 participated in the regulation of IBI.** Downregulated miR-23b mediated by transcription factor c-Myc alleviates IBI *via* Nrf2 upregulation.

Taken together, the findings from the present study suggest that downregulated miR-23b mediated by transcription factor c-Myc alleviates IBI *via* Nrf2 upregulation (Figure 6). These findings may provide a better understanding regarding the role of c-Myc in IBI. Moreover, further studies are required to provide additional evidence on whether miR-23b expression is tissue-specific or global. Prospective studies that could translate these findings regarding the role of c-Myc in IBI into clinical applications will be greatly beneficial.

## Supplementary Material

Supplementary figures and table.

## Abbreviations

IBI: Ischemic brain injury
MCAO: middle cerebral artery occlusion
OGD: oxygen glucose deprivation
miR-23b: microRNA-23b
Nrf2: Nuclear factor erythroid 2-related factor 2
