# Supplementary material for: Downregulation of miR-23b by transcription factor c-Myc alleviates ischemic brain injury by upregulating Nrf2
Source: Int J Biol Sci. 2021 Aug 26;17(13):3659–71. doi: 10.7150/ijbs.61399 (PMC8416714; doi:10.7150/ijbs.61399)
Supplement: Supplementary file 1 — Supplementary figures and table. [file ijbsv17p3659s1.pdf]

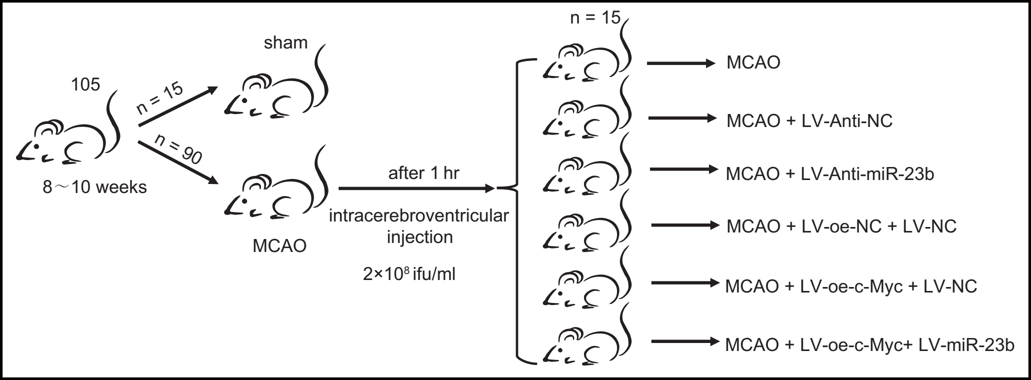
105
8～10 weeks
n = 15
n = 90
sham
MCAO
after 1 hr
intracerebroventricular
injection
2×10⁸ ifu/ml
n = 15
MCAO
MCAO + LV-Anti-NC
MCAO + LV-Anti-miR-23b
MCAO + LV-oe-NC + LV-NC
MCAO + LV-oe-c-Myc + LV-NC
MCAO + LV-oe-c-Myc+ LV-miR-23b

Figure S1. Experimental flow chart of IBI mouse model.

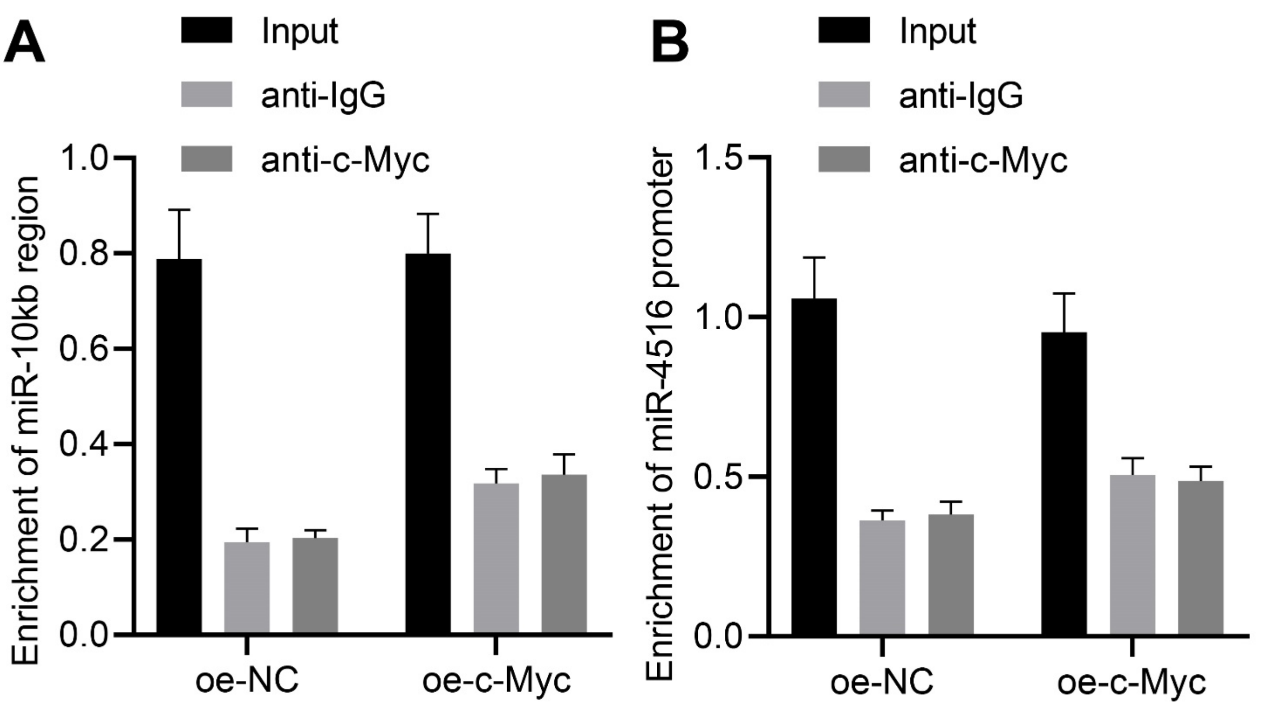

Figure S2. The enrichment of c-Myc in miR-23b and miR-4516. A, Enrichment of the transcription factor c-Myc in a 10 kb region in upstream of miR-23b assessed by ChIP. B, Enrichment of the transcription factor c-Myc in the promoter of miR-23b evaluated by ChIP.

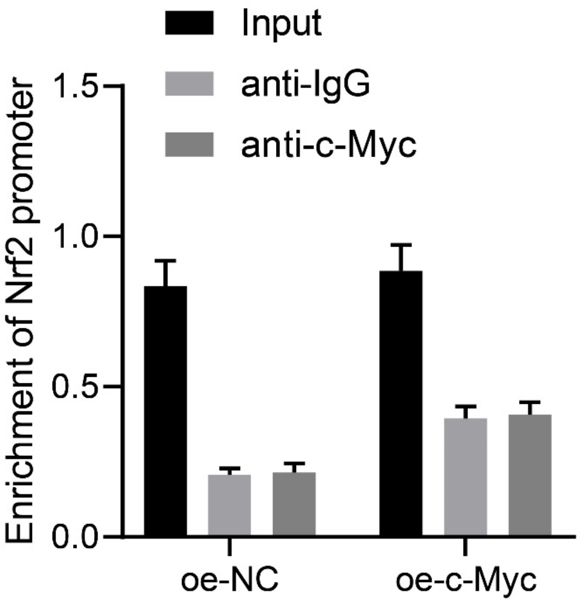

Figure S3. Enrichment of the transcription factor c-Myc in Nrf2 promoter assessed by ChIP.

Table S1 RT-qPCR primer sequence

	Primer sequences (5'-3')
hsa-miR-23b	F: ACACTCCAGCTGGGATCACATTGCCAGGGAT
	R: CTCAACTGGTGTCGTGGAGTCGGCAATTCAGTTGAGGTGGTAAT
mmu-miR-23b	F: CCAAAAGGGACCGAA
	R: TGTCGTGGAGTCGGC
mmu-Nrf2	F: TTGGCAGGAGCTATTTTCC
	R: GAACAGCGGTAGTATCAGC
hsa-U6	F: CGCTTCGGCAGCACATATAC
	R: CAGGGGCCATGCTAATCTT
mmu-U6	F: GCATGACGTCTGCTTTGGA
	R: CCACAATCATTCTGCCATCA
mm-GAPDH	F: AGGTCGGTGTGAACGGATTTG
	R: TGTAGACCATGTAGTTGAGGTCA

Note: RT-qPCR, reverse transcription quantitative polymerase chain reaction; miR-23b, MicroRNA-23b; Nrf2, Nuclear factor erythroid 2-related factor 2; GAPDH, glyceraldehyde 3-phosphate dehydrogenase; F, forward; R, reverse.
